# Physiological and Pathological Roles of RAD52 at DNA Replication Forks

**DOI:** 10.3390/cancers12020402

**Published:** 2020-02-10

**Authors:** Eva Malacaria, Masayoshi Honda, Annapaola Franchitto, Maria Spies, Pietro Pichierri

**Affiliations:** 1 Mechanisms, Biomarkers and Models section, Department of Environment and Health, Istituto Superiore di Sanità, Viale Regina Elena 299, 00161 Rome, Italy; evamalacaria@gmail.com (E.M.); annapaola.franchitto@iss.it (A.F.); 2 Department of Biochemistry, University of Iowa Carver College of Medicine, 51 Newton Road, Iowa City, IA 52242, USA; masayoshi-honda@uiowa.edu

**Keywords:** RAD52, replication fork reversal, replication fork recovery, genome stability, target therapy

## Abstract

Understanding basic molecular mechanisms underlying the biology of cancer cells is of outmost importance for identification of novel therapeutic targets and biomarkers for patient stratification and better therapy selection. One of these mechanisms, the response to replication stress, fuels cancer genomic instability. It is also an Achille’s heel of cancer. Thus, identification of pathways used by the cancer cells to respond to replication-stress may assist in the identification of new biomarkers and discovery of new therapeutic targets. Alternative mechanisms that act at perturbed DNA replication forks and involve fork degradation by nucleases emerged as crucial for sensitivity of cancer cells to chemotherapeutics agents inducing replication stress. Despite its important role in homologous recombination and recombinational repair of DNA double strand breaks in lower eukaryotes, RAD52 protein has been considered dispensable in human cells and the full range of its cellular functions remained unclear. Very recently, however, human RAD52 emerged as an important player in multiple aspects of replication fork metabolism under physiological and pathological conditions. In this review, we describe recent advances on RAD52’s key functions at stalled or collapsed DNA replication forks, in particular, the unexpected role of RAD52 as a gatekeeper, which prevents unscheduled processing of DNA. Last, we will discuss how these functions can be exploited using specific inhibitors in targeted therapy or for an informed therapy selection.

## 1. Introduction

A coordinated network of proteins surveys the genome in human cells and ensures maintenance of its stability [1]. Since complex genomes, like the human one, are particularly vulnerable during their duplication, the safe handling, processing and recovery of perturbed replication forks is critical to prevent the accumulation of DNA damage and to ensure genome stability [2,3,4,5,6]. Moreover, avoidance of DNA damage in S-phase is also critical to prevent the so-called replication stress, which is one hallmark of cancer [7,8,9]. Although replication stress is recognized as one of the fuels propelling cancer transformation and proliferation it is also its Achilles’ heel; it is therefore both a target and a biomarker for possible therapeutic interventions [10]. Hence, a detailed knowledge of the interconnected molecular processes involved in ensuring timely and complete DNA replication is essential to our ability to understand maintenance of genome integrity and to pursue novel anticancer therapies.

Human RAD52 protein has recently emerged as an important player in several mechanisms that promote cellular viability while increasing genome instability [11,12]. While Rad52 is critical for all known homology-directed DNA repair mechanisms in yeast [11,13,14], mammalian RAD52 was previously considered to be dispensable for recombination-based DNA repair and its knock out in mouse does not show any lethality or other remarkable phenotypes as those associated with the RAD51 knock out [15,16]. Based on the studies in chicken DT40 cells [16,17], it has been proposed that the vertebrate RAD52 may be involved only in a limited subset of the recombinational DNA repair events. Indeed, the most crucial recombination mediator function of yeast Rad52 is played by BRCA2 tumor suppressor protein in higher eukaryotes, including mammals, fungi, and plants [18,19,20].

Remarkably, much interest in RAD52 function arose since its deficiency has been shown to be synthetic lethal with biallelic mutations in or absence of *BRCA1/2* or *BRCA*-related genes [13,21,22,23], which are found mutated or de-regulated in many hereditary and sporadic breast and ovarian cancers [24,25].

While biochemical features of RAD52, its more novel double-strand break (DSB) repair functions and its modifications are the focus of other reviews in this issue [12,26,27,28], here, we will discuss the unexpected roles that RAD52 plays at the DNA replication fork and their implications for cancer therapy.

## 2. Role of RAD52 in DSBs Repair

Replication stress often generates DSBs that must be correctly repaired to ensure genome stability. The consequences of aberrant DSBs repair can cause chromosomal aberrations, DNA rearranges and mutations followed by cancer onset [29]. DNA double strand breaks may be caused by endogenous sources such as byproducts of metabolism and DNA replication–transcription conflicts, or may derive from treatments with drugs or chemotherapeutic agents [1]. To maintain genome integrity and to ensure continuity of the replicating chromosomes, cells have developed several pathways to respond to DSBs: Non-homologous end-joining (NHEJ), homologous recombination (HR), single-strand DNA annealing (SSA), and microhomology-mediated end joining (MMEJ). NHEJ, is prone to introduce errors since it re-ligates ends of DNA independently from the sequence context; as a consequence, rejoining can involve ends originating from different DSBs leading to chromosome rearrangements [1,30,31]. In HR, genetic information is maintained correctly as one sister chromatid is used as a template, although HR is not necessarily error-free [31]. DNA ends, to be repaired by HR, must be processed to produce single stranded DNA (ssDNA), which is coated by the ssDNA binding protein Replication Protein A (RPA) and then by RAD51 recombinase, which carries out the search for and invasion of homologous sequence. The assembly of the RAD51 nucleoprotein filament, which is the active species in HR, is assisted by recombination mediators and various RAD51 paralogs that help RAD51 to properly load and compete with RPA at ssDNA [31]. In yeast, Rad52 is the main recombination mediator, but in human cells this function is carried out by BRCA2 [31]. This implies that RAD52 may play only minor roles in HR in human cells, possibly substituting for BRCA2 under some circumstances [22,23,32]. The most obvious explanation to the RAD52-BRCA2 synthetic lethal relationship is that RAD52 takes over BRCA2 as recombination mediator when it is absent. Although RAD52 seems responsible for some of the RAD51 focus forming activity [32], it is clearly unable to prevent HR defects in BRCA2 deficient cells [33]. Moreover, loss of RAD52 is synthetic lethal also with that of BRCA1 [23,34]. BRCA1 plays many of its functions before strand-invasion [35] suggesting that the mechanism behind this genetic interaction might correlate with multiple functions of RAD52.

Notwithstanding the minor role in HR, RAD52 plays a crucial role in the single-strand annealing pathway of repair (SSA) [36] given its ability to bind RPA-coated ssDNA and promote its reannealing [37,38]. The key function of RAD52 in SSA has been proposed to explain synthetic lethality between RAD52 depletion and BRCA2 defects. According to this hypothesis, end-resected DSBs left unrepaired in BRCA-deficient cells can be sealed using RAD52-dependent SSA. SSA, however, is a mutagenic process as only homologous sequences can be annealed. While human genome is laden with repetitive sequences that can provide homology for SSA, the information between the repetitive sequences surrounding the DSB is irreversibly lost. Like NHEJ, SSA can also lead to joining the ends that belong to different chromosomes. Another process which has been recently demonstrated to involve RAD52 is MMEJ, which is another specialized DNA repair pathway that can act on resected DNA ends [39]. Similarly to the role in SSA, RAD52 has been shown to promote annealing of regions with >50 nt of homology especially in genomic regions flanked by extensive non-homologous sequences [39]. As for SSA, also the function of MMEJ could be relevant to back-up the severe HR defect shown by BRCA1/2 deficient cells [39]. 

**Figure 1:**
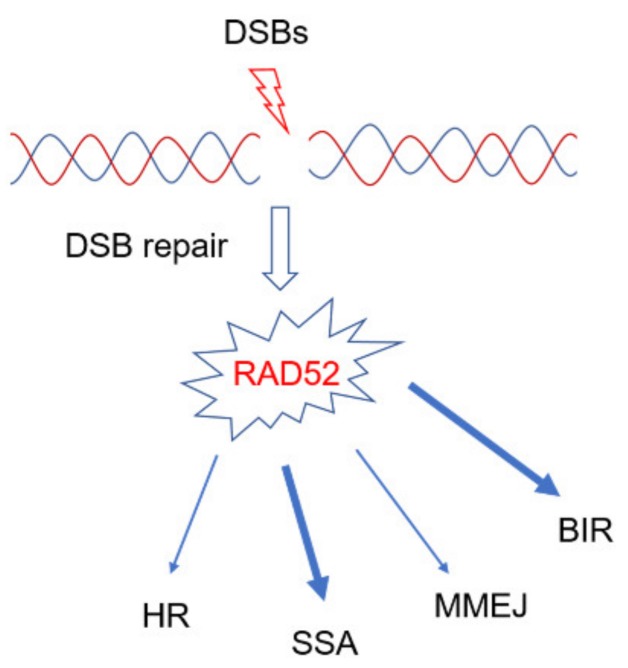
RAD52 is involved in multiple DNA repair pathways activated by double-strand breaks (DSBs). RAD52 is involved in different DSBs repair pathways. Mainly, RAD52 plays essential functions in single-strand annealing (SSA) or break-induced replication (BIR). MMEJ: Microhomology mediated end-joining; HR: Homologous Recombination.

Interestingly, all the above mentioned DSBs repair pathways involving RAD52 (Figure 1) work at two-ended DSBs but are less-likely to be able to deal with one-ended DSBs, which occur during replication because of collapse of the replication fork [40]. One-ended DSBs are likely the predominant type of DNA lesion accumulating in cells defective of BRCA1/2 and even in cells treated with PARP inhibitors since inhibition of PARP hampers cell’s ability to repair the single-strand breaks (SSBs) before S-phase [37] and attempts to replicate through unrepaired SSBs inevitably degenerate into one-ended DSBs at the fork.

Hence, a specific function of RAD52 at the replication fork is likely to be the most physiologically relevant and critical for the genetic relationship of synthetical lethality with BRCA1/2.

One-ended DSBs can be repaired by the break-induced replication process (BIR) [41]. Similar to HR, BIR starts with DSB resection, the RAD51 nucleoprotein filament formation and the strand invasion, but the invasion can be followed by a D-loop migration that results in asynchronous synthesis of the leading and lagging strand, possibly up to the chromosome end (for a review see: [41,42]). Since BIR is engaged when each end of the DSB acts independently, DSB is probably the preferred recombination pathway used to repair one-ended DSBs at replication forks. In yeast but also in human cells, BIR may involve RAD51 or RAD52 [42,43,44], however, human RAD52 has been recently found to be critical for repair by BIR of collapsed forks formed after oncogene activation or abrogation of BRCA2 [45,46]. Thus, RAD52 has been observed to be able to promote the restart of collapsed replication forks.

Interestingly, the ability of RAD52 to perform BIR has also been observed during mitosis in a process termed MiDaS (mitotic DNA synthesis) that ensures the correct replication of fragile sites and of the DNA regions that are difficult to replicate [47]. Similarly, recent studies have demonstrated that RAD52 is involved in the alternative maintenance of telomeres (ALT) preventing cellular senescence through BIR pathway [48,49,50]. Since cancer cells are often characterized by ALT phenotype, overexpression of RAD52 might correlate with capability to proliferate in uncontrolled manner.

## 3. Regulation of RAD52 and its Relevance in DSB Repair

Given the possibility that RAD52 participates to multiple DNA repair pathways activated by DSBs, it is expected that its function in each of these pathways is regulated or controlled by specific post-translational modifications or protein–protein interactions. Indeed, several of the factors involved in DSBs repair, either two-ended or one-ended, are regulated by phosphorylation [51]. At least one, functionally-relevant post-translational modification has been identified in RAD52 [52]. Phosphorylation of RAD52 by the c-ABL kinase at Tyrosine residue 104 has been demonstrated to affect RAD52 ability to perform SSA [52]. Phosphorylation by c-ABL also promotes RAD52 foci formation after DSBs [53,54]. Since c-ABL is activated by DNA damage and found overexpressed in a subset of solid cancers [55,56], it is tempting to speculate that tumors with elevated levels of c-ABL might be more resistant to therapies inducing DNA damage. Beside the reported phosphorylation by c-ABL, RAD52 can be modified by SUMOylation, and this could modulate the ssDNA annealing activity of RAD52 promoting gene-conversion [57,58]. Similarly, RAD52 interaction with specific partners is expected to modulate its repair function. It has been very recently reported that association of RAD52 with DSS1 can promote BIR in cell models [59]. RAD51AP1 also interacts with RAD52 and facilitates its recruitment to telomeres during ALT [60]. However, other functional clues on how RAD52 can be regulated are missing and deserve investigation.

## 4. Replication Fork Recovery

DNA replication forks are often perturbed by lesions that hinder replication [61]. In addition, spontaneous formation of secondary DNA structures and RNA transcription can also block progression of the replisome [61]. Since faithful completion of genome duplication is essential for cell viability and genome integrity, cells, and especially the cells of higher eukaryotes, have multiple mechanisms to restart DNA replication [4]. In mammalian cells, the possibility to overcome blocked replication forks by passive replication through dormant origins represents a valid option [62]. However, when passive replication or repriming downstream the blocked replication forks cannot be used, cells need to “repair” the stalled replication fork and resume its function. This situation typically occurs when cells are treated with agents blocking replication fork progression, such as hydroxyurea or aphidicolin, or upon treatment with many anticancer agents, such as topoisomerase inhibitors. Possibility to exploit neighboring dormant origins to complete duplication past the blocked replication fork (i.e., restart by passive replication), however, may be a concern when dormant origins are absent or are very few, such as in a subset of common fragile sites [3,62,63].

In recent years, it has been demonstrated that efficient replication fork recovery in mammalian cells involves remodeling of the forked DNA structure: The so-called fork reversal reaction [64]. Replication fork reversal results in regression of the fork and reannealing of the two nascent strands, which leads to the formation of a Holliday junction-like structure (colloquially referred to as a “chicken foot structure”) [65]. Replication fork reversal has three beneficial effects: 1) It protects ssDNA formed at the blocked fork; 2) it produces an intermediate that might also be used to restart replication using recombination; 3) it provides an opportunity for the template switching, which puts the lesion behind the fork and thus bypasses the roadblock [65,66,67].

Fork reversal can be carried out by several proteins in human cells including SMARCAL1, ZRANB3, HLTF, and RAD51 [68,69,70,71,72] although many other proteins, such as WRN, FANCM, or RAD54, can perform fork reversal in vitro [73,74,75]. It is not yet completely understood if these proteins act in a linear pathway, or if they are recruited in response to specific “roadblocks”. Of note, while SMARCAL1 is recruited at parental gaps formed in the leading strand through RPA, ZRANB3 loads through association with PCNA most probably at the lagging strand [76,77,78]. A recent work suggested that all the factors implicated in fork reversal *in cellulo* act sequentially [79] but how they share the job at the stalled forks, or the precise recruitment timing and regulatory events are still missing pieces of this fascinating puzzle.

Once reversed forks are formed, they need to be stabilized against degradation because the reannealed arm of the reversed fork structures (i.e., the middle toe of the “chicken foot”) contains a free DNA end that resembles a DSB. Stabilization of the reversed fork is a process dependent on BRCA2-RAD51 axis, but also requiring additional factors [80,81]. Loss of fork stabilization factors inevitably leads to degradation of nascent strand that may extend up to several kilobases away from the fork [66]. Pathological degradation of the reversed fork occurs by the coordinated action of two exonucleases, MRE11 and EXO1 [46]. Indeed, in BRCA2-deficient cancer cells, unprotected regressed arms become the entry point for MRE11, whose recruitment is performed by RAD52, which binds MRE11 [80,82].

Exposure of nascent strand DNA due to reversed fork can be also observed under normal handling of stalled forks and is linked to replication fork recovery possibly by invading back the reannealed template (i.e., by recombination). This “normal” resection of the 5’-protruding end of a reversed fork is performed by DNA2 in cooperation with the WRN helicase similarly to what happens at DSBs [83,84]. As indicated from yeast studies, processed nascent strands of reversed forks can also be used as an intermediate in the template switching mediated error-free lesion bypass [67].

Of note, although RAD51 has been involved in assisting fork reversal and in fork protection, only the latter requires BRCA2 [69]. One possibility is that only few molecules of RAD51 are needed to help fork reversal bypassing the requirement of BRCA2 or that other mediators, such as RAD51 paralogs, can assist RAD51 loading at parental ssDNA. Indeed, a Rad51 paralog-containing Shu complex has been recently implicated in the lagging strand abasic site tolerance in yeast [60]. Similarly, a BRCA2 separation-of-function mutant-BRCA2 S3191A-suggests that its role in HR is different from that carried out during fork protection [80].

Loss of fork protection induced by mutations or depletion of BRCA2 may also result in the formation of DSBs at forks [46,80,85,86]. Although many endonucleases, such as XPF or GEN1, can target recombination/replication intermediates in S or G2 phase upon replication stress [87,88], fork breakage occurring downstream of fork degradation by MRE11 involves the endonuclease activity of the MUS81 complex to cleave at 5’-flaps [46]. Formation of DSBs positively affects ability to restart perturbed replication forks in absence of BRCA2 but undermines chromosome integrity [46].

Although, at least in vitro, fork restoration (i.e., the back reaction of fork reversal) maybe carried out by at least two of the factors acting at reversed fork: SMARCAL1 and WRN [76,89], in cellulo, fork restoration seems to be dependent on the RECQ1 helicase activity under the control of PARP1 [90].

## 5. RAD52 as “Gatekeeper“ of Perturbed Replication Fork

**Figure 2:**
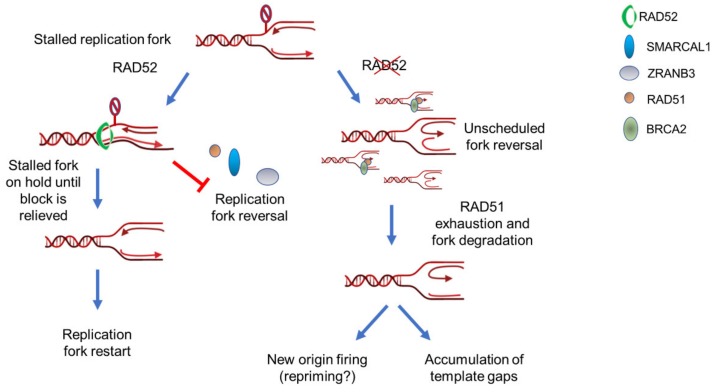
RAD52 is a gatekeeper that limits excessive replication fork reversal. Upon fork stalling, RAD52 can associate with parental ssDNA at fork. Binding of RAD52 at fork can induce a rearrangement of the DNA closing the Y structure to counteract recruitment of fork reversal enzymes such as SMARCAL1 or ZRANB3. This gatekeeper function probably put the stalled fork on hold until it is ready to restart. If RAD52 is absent or its association with ssDNA is abrogated, the recruitment of fork reversal enzymes is abnormally increased leading to exhaustion of fork protection factors, such as RAD51. Such exhaustion of fork protection factors triggers degradation by MRE11 and eventually leads to pathological fork recovery and genome instability.

Most of the roles proposed for RAD52 at the replication fork so far concerned pathological conditions. Either RAD52 acts to repair DSBs by SSA or MMEJ or it acts upon collapsed replication forks to perform BIR [12]. However, RAD52 has a remarkable affinity for ssDNA and the ssDNA-RPA complex, which is crucial intermediate in all reactions taking place at perturbed replication forks, and such ssDNA-binding activity is essential for promotion of MUS81-dependent cleavage [85]. Recently, RAD52 was also shown to be involved in protecting stalled replication forks under non-pathological conditions [91]; that is without any other mutation or defect inducing replication fork demise, such as checkpoint deficiency, oncogene activation or BRCA2 loss. In a wild-type background, RAD52 protects reversed replication fork from MRE11-degradation [91]. As we outlined earlier on, MRE11-dependent degradation is a pathological response to fork deprotection following reversal of the replication fork, which is triggered by loss of BRCA2 or RAD51. How loss of RAD52, which is dispensable for RAD51 loading at reversed fork, can promote MRE11-dependent degradation? Strikingly, the absence of RAD52 or inhibition of its association with ssDNA leads to uncontrolled fork reversal that exhausts the pool of RAD51 necessary to protect nascent DNA from MRE11-dependent degradation [91]. In other words, RAD52 plays its protective role upstream fork reversal, as opposed to the well-described function of BRCA2 that occurs downstream the reversal of the replication fork [46,69,79]. RAD52 can bind the stalled replication fork before fork reversal and reconfigures the fork to prevent loading of SMARCAL1, ZRANB3, and RAD51 [91]. In this way, RAD52 plays a role of a molecular gatekeeper ensuring that fork reversal enzymes load only when needed (Figure 2). RAD52 can bind RPA and binding occurs through a RQK sequence that is the same used by SMARCAL1 [37,92]. Thus, RAD52 might also prevent fork reversal by competing with SMARCAL1 for the binding to RPA, an event that is essential for the proper function of SMARCAL1 in fork reversal [76]. The gatekeeper function of RAD52 requires its ssDNA binding ability as treatment with EGC, an inhibitor of ssDNA-RAD52 association [85], and RNAi depletion give the same phenotype [91]. However, RAD52 also binds dsDNA [93]. Whether, only ssDNA binding is sufficient to exert the gatekeeper function or also that with dsDNA or RPA are involved awaits an experimental proof.

This anti-reversal function of RAD52 is functionally equivalent to that of RADX, a RAD51 antagonist, whose loss also results in excessive RAD51 function at the fork [94,95,96]. However, while loss of RADX leads to hyperactivation of RAD51, which interferes with DNA replication and eventually generates MUS81-dependent DSBs [95], loss of RAD52 stimulates loading at replication fork of several fork reversal enzymes, including RAD51 [91].

In both cases, loss of RADX or RAD52 affects replication fork progression by slowing down its rate, although at different extents [91,94]. Indeed, depletion of RADX can reduce much more fork progression than loss of RAD52 does. Even if either inhibition of RAD52 or depletion of RADX increases new origin firing [91,96], firing of dormant origins very close to the site where the replication fork has stalled, or even repriming events might mask a more severe impairment of fork progression in cells inhibited of or depleted for RAD52 [91].

In sharp contrast with fork degradation occurring in BRCA2-defective cells or under other conditions leading to incorrect fork protection [97], loss of RAD52 or its inhibition does not stimulate a loss of both nascent strands at the arrested fork, which can be detected as shortening of replication tracks on stretched DNA fibers but determine degradation of just one of the two nascent strands [91]. Moreover, apparent degradation of just one of the two nascent strands might correlate with the higher number of parental gaps observed upon inhibition of RAD52, even under unperturbed conditions [91]. Why this occurs is unknown, but it might reflect the concomitant loss of the gatekeeper function of RAD52 and its distinct requirement for the recruitment of MRE11 at deprotected reversed forks [82]. Indeed, in BRCA2-deficient cells, RAD52 contributes to loading of MRE11 through a still undefined mechanism suggesting that, upon fork reversal, RAD52 might persist at reversed forks carrying out additional functions. This multi-tasking behavior of RAD52 fits well with the ability to find it bound to both parental and nascent ssDNA [91], which is exposed only after fork reversal [64,69,98,99]. In principle, binding of RAD52 to nascent ssDNA might correlate with a role in promoting recruitment of MRE11 at deprotected forks. Furthermore, the presence of some RAD52 at nascent ssDNA also in wild-type cells suggests that a fraction of stalled forks might undergo deprotection even in the presence of BRCA2, a possibility that deserve more investigation.

Intriguingly, recent works demonstrated that excessive fork degradation could be prevented by repriming downstream the stalled fork by Primpol [100]. This mechanism would compete with fork reversal and would contribute to restoration of fork integrity and PARPi-resistance in BRCA2-defective cells, making Primpol also a potential therapeutic target [100]. Moreover, repriming by Primpol would be also stimulated at degraded forks upon UV-induced DNA damage, which would suggest that fork reversal- and Primpol-mediated restarting mechanisms act in parallel [101]. In RAD52-deficient cells repriming or de-novo dormant firing is expected to occur at high levels suggesting that also unrestricted fork reversal and incomplete fork processing by MRE11 stimulates alternative ways to ensure replication progression. It is worth noting that pathological fork degradation by MRE11-EXO1 subsequently requires MUS81-dependent cleavage to support fork recovery [46]. As we will discuss later, RAD52 interacts with MUS81 and is essential for formation of MUS81-dependent DSBs [102]. Hence, RAD52 might protect stalled forks from excessive remodeling but, under pathological conditions, such as BRCA-deficiency, oncogene activation or checkpoint inactivation, could support fork restart through stimulation of MRE11-MUS81 axis.

Could the RAD52 action as a gatekeeper at the perturbed replication forks explain, in part, the synthetic lethal interaction between RAD52 and BRCA2 in human cells [22,23]?

**Figure 3:**
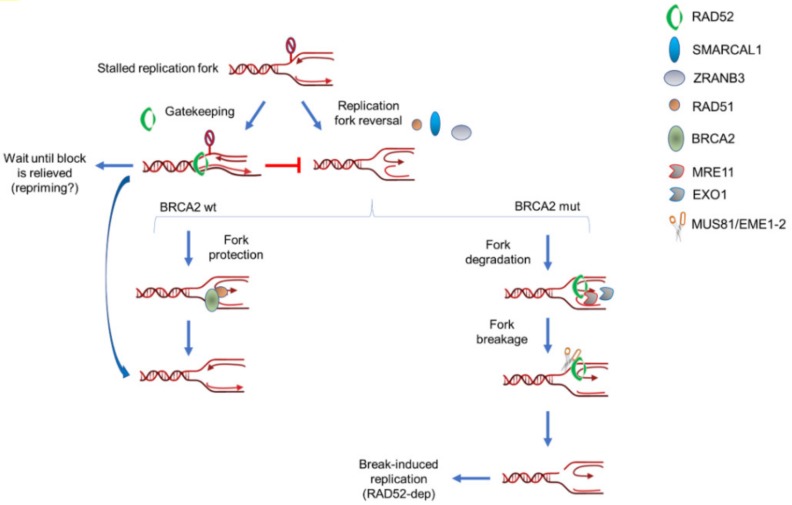
Multiple roles of RAD52 in response to normal or pathological fork stalling. RAD52 can perform multiples and sequential roles at perturbed replication forks in wild-type or BRCA2-deficient cells. Under normal conditions, such as in BRCA2 wild-type cells, RAD52 mainly functions as gatekeeper and limits unscheduled fork reversal (left arm). However, it might be also involved in MRE11 recruitment at a subset of deprotected forks. When BRCA2 is absent or mutated, RAD52 may still play its gatekeeper role but it is expected to be much more required downstream fork reversal (right arm). At deprotected forks, RAD52 is required to recruit MRE11 and initiate pathological fork degradation. After fork degradation, RAD52 would persist at degraded forks to recruit the MUS81 complex through direct protein–protein interaction. Hence, RAD52 would stimulate formation of MUS81-dependent DSBs through, at least, two independent mechanisms: recruitment of MRE11 and recruitment/stimulation of MUS81. Finally, RAD52 would promote repair of MUS81-induced DSBs through break-induced replication (BIR).

Current explanation of the BRCA2-RAD52 synthetic lethality is largely based on the function of RAD52 in RAD51-independent DSB repair, such as SSA or BIR [12]. Alternative explanation is that RAD52 cooperates with MUS81 to process reversed, deprotected forks, in BRCA2-deficient cells [85]. However, loss of RAD52 stresses the fork deprotection pathway but also calls for RAD51, and BRCA2, when forks recover to repair post-replicative gaps that accumulate in the absence of RAD52 [91]. Indeed, inhibition of RAD52 only during fork arrest and subsequent inhibition of RAD51 only during recovery results in accumulation of segregation defects and severe cell death in mitosis [91]. A condition that is not worsened if RAD52 inhibition is prolonged also during recovery [91]. Hence, loss of RAD52 might require intact BRCA2-RAD51 for viability, suggesting that the genetic relationship between RAD52 and BRCA2 is more complex than expected and challenging the current model (Figure 3).

The apparent reliance of RAD52-inhibited cells on RAD51 during recovery from even a short perturbation of fork progression might also, provocatively, explain why RAD52 knock-out is not associated to major phenotypes. Indeed, loss of the apparently relevant RAD52 function(s) in S-phase could backed-up by RAD51, ensuring proliferation.

## 6. RAD52 as a Partner of MUS81

Prolonged fork stalling or the failure of replication restart can lead to fork collapse and DSBs formation [103,104,105]. Similarly, loss of fork protection induces DSBs at forks [46,106,107,108]. Under these conditions, RAD52 has been shown to perform roles either downstream or upstream DSBs formation [45,82,102]. Several works in the last years supported or reported a function for RAD52 in the repair of collapsed replication forks by BIR [45,47,59,109,110]. In yeast, the relevance of Rad52 for BIR have been vastly demonstrated over the years as well as it has been shown that Rad52 participates to both Rad51-dependent and independent BIR [42]. In human cells, the first evidence for a critical function for RAD52 upon fork collapse came from studies in cells suffering from oncogene-induced replication stress [45,110]. Oncogene induction, such as Cyclin E overexpression, undermines fork stability and determines induction of DSBs that requires RAD52 for their repair [45]. DNA breaks formed upon oncogene induction are, as those occurring in BRCA2-defective cells in S-phase, instrumental for recovery of DNA replication although they generate chromosome instability [46,102,111]. Under other replication fork perturbing conditions, such as those induced by prolonged exposure to low-doses of aphidicolin, which is expected to sensitize common fragile site to breaks (CFS; [6]), the BIR-related function of RAD52 seems to extend from S-phase to late-G2/M-phase where it participates with DNA Polδ in MiDAS [47]. This role of RAD52 is expected to support stability of CFS, helping to counteract mitotic defects [47].

In MiDAS and BIR, through a still unclear mechanism, RAD52 may also contribute to formation of a D-loop, possibly exploiting its ssDNA annealing activity, which is then extended by the specialized POLD3 subunit of the replicative DNA Polδ [112]. In yeast, this function may be carried out in cooperation with other factors, such as Rad59 [113,114].

Notably, RAD52 acts not only to repair DSBs but also to stimulate their formation. This apparent counterintuitive function has been observed when replication fork is destabilized by checkpoint inactivation and correlates with cleavage by the MUS81 complex [102]. In this scenario, RAD52 plays a key role generating a structure, which is a substrate for MUS81 cleavage [102] paralleling that reported in yeast [114]. Although, at least in vitro, MUS81 has been shown to efficiently cleave a RAD52-assembled D-loop [102], whether this occurs also in the cell is unknown. Additionally, the role of RAD52 in the generation of MUS81-dependent DSBs has been demonstrated after depletion of BRCA2 and HU treatment [85]. In this case, it is still unknown whether RAD52 form a D-loop or rather is only involved in the recruitment of MRE11, which is mandatory for subsequent cleavage by the MUS81 endonuclease [46].

Intriguingly, upon BRCA2 depletion, the substrate supposed to be cleaved by MUS81 is a 5’-flap generated by exonucleolytic degradation at the regressed arm of the reversed fork [46]. This is a different substrate respect the hypothesized D-loop cleaved by MUS81 downstream RAD52 after checkpoint deficiency or even upon BRCA2 depletion [85,102]. This opens the possibility that either the identity of the intermediate processed by the RAD52-MUS81 duet differs under different pathological conditions or that RAD52 may play multiple and sequential functions at unprotected forks (Figure 3). When BRCA2 function is lost, RAD52 might stimulate MUS81 cleavage of the flap indirectly by promoting MRE11 loading and then could promote D-loop formation and invasion from the processed regressed arm of the reversed fork initiating BIR and finally recruit MUS81 again to cleave this intermediate. Since all this would happen in a situation in which RAD51 cannot be assembled to protect the fork—i.e., BRCA2-deficiency-it makes sense that a D-loop assembled by RAD51 is refractory to MUS81-dependent cleavage [102]. Worth noting, RAD52 is required to ensure replication fork progression under these situations although its activation greatly stimulates rearrangements being able to produce specific mutational signatures as found also in yeast cells [42,109,115,116,117,118].

It is still unknown if RAD52 promotes MRE11 loading under checkpoint inhibition and it is reasonable that finding a mutant of RAD52 that cannot interact with MUS81 or MRE11 might help clarifying the predominant substrate targeted by RAD52 in absence of BRCA2 or other fork protection factors, such as BOD1L, WRNIP1, or FA pathway [119,120,121].

## 7. Conclusions and Potential Implications of Replication Fork-Related RAD52 Roles in Cancer Therapy

In recent years, RAD52 has emerged as an attractive target for developing new anticancer therapies targeting HR deficient tumors [27]. RAD52 removal or its pharmacological inhibition is synthetically lethal with biallelic defects in genome caretakers BRCA1, BRCA2, or PALB2 [23,85,122,123,124]. While this situation parallels synthetic lethality between BRCA defects and inhibition of PARP1, the mechanisms of synthetic lethality are likely different and therefore inhibiting RAD52 may be beneficial in cases where PARP inhibitor resistance has emerged [125,126]. Moreover, it has been shown recently, that simultaneous inhibition of PARP1 and RAD52 can have a synergistic effect on the tumor cell killing [127]. It remains unclear, however, which of the many cellular functions of RAD52, which we discussed above, allow for survival and proliferation of BRCA-deficient cells and whether there are other circumstances when it would be beneficial to target RAD52. Cancer cells are characterized by high genome instability and capacity to respond to replication stress through activation of pathological pathways. This pertains to cancers defective in some aspects of genome maintenance, such as BRCA-deficient tumors, as well as to cancers that are deficient in cell cycle check point and therefore have to rapidly complete their replication program [128]. Thus, understanding how to target these pathways could be used as a strategy to selectively kill tumor cells. Since alterations in mechanisms of fork remodeling had been linked to cancer onset, targeting fork reversal or fork protection could offer new options for development of cancer therapies.

Collectively, RAD52 appears at crossroads of the processing of destabilized replication forks in human cells (Figure 2 and Figure 3). Indeed, it is required to load MRE11 at destabilized reversed forks and to promote MUS81-dependent breakage by ether starting pathological degradation by MRE11 or through direct association with MUS81. Intriguingly, loss of RAD52 strongly impairs viability of checkpoint-deficient cells [102]. Since CHK1 inhibitors are being tested in clinical trials [129,130,131], combining RAD52 and CHK1 inhibition might prove a much more effective strategy to kill cancer cells, especially cancer stem cells. RAD52 might be involved in repair of the resulting, MUS81-dependent, DSBs by BIR or, alternatively, by SSA. In both cases, this pathway could be highly mutagenic [109,110,118]. Hence, RAD52 might be highly relevant for the emergence of genome instability in tumors and its frequent overexpression in tumors could correlate with its crucial function to repair DSBs at replication forks after loss of p53, one of the most frequently-mutated oncosuppressor [109,132].

As opposed to this apparently crucial function under pathological fork stalling, RAD52 acts as a gatekeeper of replication forks reversal, counteracting the activation of pathological/backup pathways driven by MRE11 because of RAD51 exhaustion [91].

RAD52 has been often found over-expressed in tumors. Since the response to PARPi partially depends on degradation of the reversed forks [133], this anti-reversal function of RAD52 might imply that its over-expression confers resistance to PARPi. This is a possibility that deserve further investigation.

On the other hand, inhibition of RAD52 partially recapitulates loss of BRCA2 in terms of fork deprotection [91]. Thus, in tumors in which BRCA2 is active, inhibition of RAD52 could be a valuable strategy to exploit PARPi. Although it has been recently reported that PARPi and RAD52 deficiency show a synergic effect on viability in BRCA2-deficient cells, inhibition of PARP or RAD52 has no consequences if BRCA2 is functional [127]. This result would suggest that loss of fork protection per se is not enough to give PARPi sensitivity, as suggested, or that also MUS81-dependent cleavage, which is absent if RAD52 is inhibited, must take place to confer PARPi sensitivity. Further studies using triple-inhibited cells are needed to test the latter hypothesis.

To take advantage of the RAD52 inhibition in cancer therapy we need to make multiple advances on several fronts. First, the new potent and specific RAD52 inhibitors need to be developed to match the efficacy of the PARP inhibitors. Second, we need to improve our understanding of the cellular circumstances beyond BRCAness [134] under which targeting of RAD52 can lead to cancer cell death and when RAD52 inhibition can be combined with other inhibitors or DNA damaging chemotherapy. Finally, we need to develop a better understanding of the mutation load in the RAD52 overexpressing and RAD52 deficient tumors to evaluate the possibility for immune checkpoint therapy.
